# Genome Editing Dilemma: Navigating Dual-Use Potential and Charting the Path Forward

**DOI:** 10.1007/s11673-024-10358-8

**Published:** 2024-07-24

**Authors:** Ana Ruxandra Badea, Oliver Feeney

**Affiliations:** 1 University of Bucharest, Faculty of Philosophy, Splaiul Independentei 204, 060024 Bucharest, Romania; 2 University of Tübingen, Ethics of Genome Editing Research Unit, Institute of Ethics and History of Medicine, Gartenstr. 47, 72074 Tübingen, Germany

**Keywords:** Bioethics, Genome editing, Security threats, Dual-use, Military, CRISPR/Cas9

## Abstract

Contemporary genome editing techniques have made genomic intervention—from microorganism to human—more accessible, easier to use, and more accurate than previous methods. We argue that, notwithstanding its merits in treating and preventing disease in humans, genome editing represents a potential threat for domestic and international security, requiring an integrated approach in regulating, detecting, preventing, and mitigating the risk of its use for malicious purposes. Despite the global regulatory ambitions of the 2021 WHO framework, we see insufficient attention given to the future prospect of dual-use genomic technology. Drawing parallels with the nuclear field, we suggest tentative practical steps for a way forward in dealing with genome editing technologies, such as: 1) adapting national (bio)security and defence strategies to include genome editing as a possible threat (with conceivable WMD potential); 2) enhancing the international dialogue on genome editing and raising the issue at the highest level; 3) working towards a global, legally binding verification mechanism; 4) tracking genome editing technologies.

CRISPR-Cas9, alongside the emergent generation of Prime and Base editing techniques (Kantor et al. 2020) and PASTE (Yarnall et al. 2023), have made genome editing more accessible, easier to use, and more accurate than previous methods of genetic intervention. So far, most genome editing efforts have focussed on treating and preventing disease in humans. Some inherited retinal diseases, causing visual impairments, are successfully treated through gene replacement therapies (Gupta and Yiu 2022). A CRISPR-based therapy for sickle cell disease has been granted approval by the FDA, the U.K.’s MHRA, and conditional marketing authorization from the EMA (Feuerstein 2023; Wong 2023; Barrie 2024). However, these positive developments only came years *after* He Jiankui conducted heritable genome editing in humans (Cyranoski and Ledford 2018; Devlin 2023). These divergent cases underline the very different scientific, ethical, and legal directions made possible by this new generation of genome intervention technology. The dual-use potential of genome editing of humans, animals, and pathogens can “cut both ways” requiring continuous scrutiny, especially the possible risks to national and international security. Such risks exceed the focus of science and ethics alone, necessitating diplomatic, military, and legal expertise to fully assess the issue and identify viable solutions.

We argue that, notwithstanding its merits, genome editing represents a potential threat for domestic and international security, requiring an integrated approach in regulating, detecting, preventing, and mitigating the risk of its use for malicious purposes. First, we outline the potential military uses while touching upon the 2021 WHO framework for the global governance of human genome editing. Despite its global regulatory ambitions, we see insufficient attention given to the future prospect of dual-use genomic technology. We assess the main challenges to regulating genome editing dual-use and draw a parallel with the nuclear field. Finally, we suggest tentative practical steps for a way forward in dealing with genome editing technologies.

## Current and Potential Military Genome Editing Uses

Many technologies—from space technology to nuclear energy to biological and chemical agents—that harbour the potential for peaceful purposes as well as contrasting objectives are referred to as “dual-use” (Harris 2016). Genome editing technologies have similar potential to be employed for both civilian and military applications. This dual-use potential is recognized in the 2021 Report by the WHO Expert Advisory Committee that focussed on developing global standards in the oversight and governance of human genome editing (WHO 2021a). The report notes that there are “potential dual-use applications; for example, human genome editing to give resistance to chemical pollutants or to radiation for space travel could also have military applications with respect to resistance to chemical or nuclear weapons” (WHO 2021a, 7). Unfortunately, even though “dual-use potential” and “possible miliary uses” were considered sufficiently important to mention in the report, they were infrequently mentioned (WHO 2021a, 7, 26, 38, 59). This lacuna is stark given the growing literature and recent developments in dual-use potential of human genomic technology outlined below. The case for making the dual-use nature of human genome editing more central, is also strengthened by the 2024 European Commission white paper^1^ proposing reform to boost dual-use research with a greater focus on defence as well as civil objectives. While this white paper does not refer to genome editing, it can be seen to further normalize the acceptance of an overlapping and interconnected regulatory environment for dual-use technologies. Ultimately, this could someday include genome editing, in turn entailing decisions on creating red lines between permissible uses and more problematic forms.

The United Nations warns that genome editing in pathogens “could be used to develop more effective biological weapons” (United Nations 2018, ¶9). The concern refers to increases in the “lethality, duration, or ease of transmission of microbiological agents” (Kosal 2020, ¶2) or new “designer diseases” (Wickiser et al. 2020). One concern of “precision in mass destruction” is where genome editing tools may be used to target a specific ethnic or generational trait (Fatollahi and Zeinoddini 2023; Werner 2019). Such genetically edited pathogens can potentially be used to attack a population, agriculture, or the wider ecology (Esvelt and Millett 2017). In 2017, U.S. Defense Advanced Research Projects Agency (DARPA) initiated a programme called *Insect Allies* to develop “countermeasures against potential natural and engineered threats to the food supply” (DARPA n.d., ¶1), by genetically modifying viruses to be spread by insects to alter plant genes (Tucker 2018a, b). DARPA’s programme was criticized for its dual-use potential to “develop biological agents for hostile purposes and their means of delivery” (Reeves et al. 2018, 35). With CRISPR, biological weapons can become easier and cheaper to produce, and therefore, more readily available than any other type of weapon of mass destruction (WMD). It would not be implausible to think that similar genome editing activities for military purposes are already taking place on the human genome. In 2016, a doctoral researcher from China’s Academy of Military Medical Science published a dissertation entitled *Research on the Evaluation of Human Performance Enhancement Technology*, which characterized CRISPR-Cas9 as one of three primary technologies that might boost troops’ combat effectiveness (Kania and Vorndick 2019). According to some, China may have commenced human testing in order to produce a military with enhanced capabilities (Ratcliffe 2020). In 2020, U.S. army medical scientists started working on a gene therapy which “can potentially protect humans against G-type chemical warfare nerve agents for several weeks to months” (Betapudi et al. 2020, 1).

Soldiers with their genomes edited might not be supersized, bullet-proof warriors with superhuman strength. It can be as subtle as genetically editing the BHLHE41, NPSR1, ADRB1 genes to enable short sleep duration or the NTRK1, SCN9A, FAAH-OUT genes to reduce pain sensitivity or the PDE4B gene to reduce anxiety (Church 2023). There may be a genetic aspect to Post-Traumatic Stress Disorder which, if edited, potentially could be a treatment for returning soldiers but also as a psychological enhancer, enabling them to fight for longer (Cornelis et al. 2010). Green and Master point out that effective vaccinations might be hard to develop in the context of the emerging biological threats, and the best way forward might be editing of soldiers to lower susceptibility to such pathogens (Greene and Master 2018). These changes can have an overall impact on how a war proceeds. One could argue that future wars will be from behind computers and that physical engagement will no longer be necessary, reducing the importance of physical enhancement—genetic or otherwise. First of all, we are not there yet, and if there is anything that the latest wars in the Middle East or Ukraine have shown us is that land invasion and physical presence still lie at the core of military strategies. Secondly, admittedly to different degrees, the soldier sitting behind the screen would benefit as well as the soldier on the ground, if genome editing would render him calmer, more astute, and impervious to tiredness.^2^

### Treatment Versus Enhancement in a Military Context

It may be noticed here that the treatment-enhancement distinction is not doing as much work as might normally be expected. Some would view enhancement to be a form of “abuse by definition” (Feeney 2019, 236). Others would consider some health-related enhancements to be closer to therapeutics in essential normative respects. For instance, Article 13 of the Oviedo convention states that an “intervention seeking to modify the human genome may only be undertaken for preventive, diagnostic or therapeutic purposes” (Oviedo Convention 1997, under “Art. 13”). This undifferentiated approach in the military may be of ethical and regulatory concern. For example, Greene and Master point out that the U.S. currently has no regulation in place prohibiting the enhancement of army personnel for military purposes, such as performance optimization and biomedical enhancement (Greene and Master 2018; Land 2010; Jonas et al. 2010). This has the potential to translate into ethical issues such as the unknown possible off-target effects, biased or deficient informed consent, social pressure (from fellow soldiers or superiors), fairness in combat (which may eventually lead to a change in *jus in bello* and a revisiting of the international humanitarian law).

Preventative measures, such as “resilience” and “protection,” or therapeutic measures such as reduction in anxiety, mingle alongside more explicit enhancements in wakefulness, pain desensitization, and so on. In one respect, they all tend toward increasing the chances of survival and success of one side over the other. However, there seems *something* importantly different between a defensive use of genomic interventions (e.g.: protecting soldiers from harm) versus a more offensive use (e.g.: improving physical strength or other capacities of the soldier, especially to kill). The point here is to highlight two things with regard to human genome interventions: 1) the treatment (prevention) versus enhancement distinction—even if one accepts it as fundamentally sound—does not offer clear guidance in every context, and 2) the context of military use of both treatment (prevention) and enhancement is one of concern across the board, even if one also holds that the treatment/defensive use is more justifiable than the enhancement/offensive side.

## International Statements and Approaches vis-a-vis Genome Editing Military Uses

While there is no actionable intelligence on current developments in HGE activities such as “the super soldier,” there are signs that genome editing technologies have become a major interest in terms of their military potential. As of December 2018, China had made 858 CRISPR patent applications compared to 872 in the United States and 186 in Europe (Cohen and Desai 2019). An increasing number of CRISPR trials has been observed occurring in People’s Liberation Army’s medical centres across China (Kania and Vorndick 2019). The prospect of military-related genome editing not only of humans, but also non-human animals and pathogens, was introduced by the U.S. Director of National Intelligence (DNI) in their threat assessment report, and categorized as having the potential for weapons of mass destruction (DNI 2016). The U.S. Defense Threat Reduction Agency (DTRA) deems biology “a new domain of warfare” and recognizes the need to prepare for the eventuality that bio-tools are used against the country (DTRA 2022). This means that the dual-use potential of genome editing is now one of the key priorities of both the U.S. intelligence and military community. While the United States is vocal on the issue of defence against genome editing, it can be speculated that it might also be active on the offensive side, through its military R&D.

The aforementioned European Commission white paper is not the first or only focus on developing the miliary products from a dual technology, although it serves to highlight how the idea is increasingly normalized as a legitimate socio-political and economic strategy for European and Horizon funding. NATO has already gone public with its intention to integrate emerging and disruptive technologies, including biotechnologies and human enhancement (although it does not mention HGE specifically), by setting up the Defence Innovation Accelerator for the North Atlantic (DIANA), aimed at start-ups that can offer “deep tech with dual-use solutions for the Alliance” (DIANA n.d., under “About”). To further the research and development efforts, in August 2023, the Alliance announced the establishment of the NATO Innovation Fund (NIF), a multi-sovereign venture capital fund set up to stimulate and support deep tech innovation, including biotechnology (NIF n.d.). While both NIF and DIANA claim to follow NATO’s Principles of Responsible Use (NIF n.d.), it might be worth considering how such undertakings could lead to a new arms race between nations or alliances involving HGE activities, particularly regarding human enhancement.

## Challenges to Regulating Dual-Uses of Genome Editing

The information available in the public domain with regard to military use of genome editing tools can only show us a fraction of the bigger picture as to how genome editing is changing the priorities and strategies of states. Like nature, international relations abhor a vacuum, and a gap in genome editing oversight will very likely and relatively quickly be filled with the dubious ambitions of state and non-state actors. The weaponization of genome editing could entail not only military use (and abuse) by states (whose behaviour is possible to supervise and predict), but also by underground factions and terrorist groups.

A major security concern is that genome editing can evade all current measures of non-proliferation. The international governance framework which should be regulating biological activities, namely the Biological and Toxin Weapons Convention (BTWC), is well recognized for its lack of control over states’ biological stockpiles and endeavours. Combined with the lack of a harmonized global governance of genome editing, we run the risk of delving into some very murky waters which could see the naissance of “DIY bio-labs” used not solely for “fun,” but also with malicious intent, by groups or private individuals (Wickiser et al. 2020). Unsurprisingly perhaps, the main actors against a BTWC verification mechanism are the United States and China, the two leading countries in the field of biotechnology.

However, the BTWC only covers biological agents such as existing or *de novo* viruses, bacteria, fungi, and toxins. Even if it were a reliable regulatory framework with a functioning verification mechanism, there would still be the matter of human and non-human animals.

### A Parallel Between Genome Editing and Nuclear Non-Proliferation

In the absence of a functioning BTWC or any other harmonizing and legally binding framework for genome editing, nuclear non-proliferation could be seen as a source of inspiration (Kosal 2020; Wickiser et al. 2020). After all, it is a remarkable example of how the international community has managed to put aside most differences and come together under the auspices of the International Atomic Energy Agency (IAEA) to develop and implement a regulatory framework with its very own verification mechanism. There are, of course, exceptions such as India, Pakistan, and Israel (which are nuclear states *de facto* but not *de jure*), or Iran and North Korea (which developed military nuclear programmes illegally), but all in all, most nuclear activities are strictly controlled. Not even a nuclear test would nowadays escape undetected, due to the International Monitoring System of the Comprehensive Nuclear-Test-Ban Treaty Organization (CBTO). Nevertheless, there are fundamental differences between the nuclear field and genome editing activities. To begin with, military grade nuclear materials such as uranium and plutonium cannot be easily found in nature, unlike biological material, which is much easier to acquire. While nuclear and radioactive material can be safely stored under IAEA safeguards, biological material cannot be controlled and tracked unless it already exists in a laboratory. Secondly, bio-related equipment and expertise are widely available (Ostfield 2009). Not only laboratories can easily access such equipment but individuals too can acquire it, as in a DIY bio-lab for some DIY biology, also referred to as biohacking (DiEuliis and Giordano 2018). Furthermore, the same knowledge that is transmitted through scientific journals and other information-sharing platforms, and which lies at the core of scientific advancement, can also turn against us by placing valuable know-how in the hands of malicious actors.

## Possible Steps for a Way Forward

Despite the dual-use potential and danger of genome editing, there have been very few proposals and initiatives to address the issue, at societal, academic, or political levels. Given the speed of genome editing developments noted at the outset, the next decade will likely see an increase in the use and possible abuse of genome editing technologies by various stakeholders for various purposes (Kania and Vorndick 2019; Wickiser et al. 2020; Werner 2019). Therefore, the transnational nature of any biotechnology research and development (Kosal 2020) and the proliferation potential (Ostfield 2009) stand as further reasons for the regulation of genome editing in a harmonized, international governing framework. But this is not the responsibility of scientists alone, and a non-binding consensus in the scientific community, as it is now the case, would not address its overarching implications. An effective regulation can only happen if the issue is addressed by states at international level, a recommendation which the WHO advocates for human genome editing overall. However, dual-use development will likely be a leading challenge that needs to be met by the international community, and the focus of developing global standards of governance and oversight for human genome editing will increasingly encounter this issue in the near future. Given the dual-use proposals from the European Commission, and the focus on military *as well as* civilian goals being advanced for Horizon Europe funding eligibility, this may signal a potentially significant development of a new ethos (and supportive socio-political context) regarding the acceptability of combining civil and military goals. Insofar, as such dual-use technology and a supportive context will continue to develop, it would be surprising if genome editing were not included in due course. So, there is a case for challenging the very short and perfunctory discussion of the dual-use potential of human genome editing technology in the 2021 WHO report; and a case for calling for a significant advance of this discussion as a prominent addition to the WHO’s case for the development of a global governance and oversight framework addressing genome editing. To this end, we would suggest the following practical responses can be proposed for such a discussion:

1. National (bio)security and defence strategies

At national level, states need to adapt their domestic (bio)security and defence strategies to include genome editing as a possible threat (with conceivable WMD potential). Threat-awareness and risk-assessment are the first steps towards building a comprehensive (bio)security framework, which can later be backed up by mechanisms of detection, prevention and response to malicious acts involving genome editing activities.

Similarly, defence alliances such as NATO, should match their research and development efforts with robust bio-surveillance strategies and military policies, perhaps even setting up a working group focused on human genome editing technologies.

2. Enhanced dialogue

At the international level, it is necessary to broaden the dialogue on genome editing horizontally and vertically: horizontally by enhancing the dialogue between relevant national agencies, and vertically, by elevating the issue of HGE above the technocratic level and bringing it to ministerial, even presidential level.

Ideally, a meaningful conversation on the topic can be extended on a domestic level to include state-owned and private laboratories, health departments, policy makers, academics, intelligence services, military, and other national security agencies. This should be combined with a sustained dialogue at an international level which ought to include high-level state representatives. The objectives of this combined two-way dialogue are (1) to raise awareness on genome editing and facilitate a meaningful, inter-agency dialogue, and (2) to take stock of national positions of states on issues of interest regarding genome editing, which is necessary to map out obstacles/ disagreements/ polarization at state level, while increasing transparency.

3. Verification mechanism

Unlike in the nuclear field, where all activities involving nuclear and other radioactive materials must be overseen by the IAEA, full control over genome editing activities might not be possible. But one valuable lesson learned from the nuclear domain is that verification is of paramount importance, so international efforts should focus on working towards a global, legally binding verification mechanism. It is worth noting the nuclear domain, particularly the IAEA, which serves as a watchdog for the NPT. Equally important lessons are to be learnt from the BTWC which has no verification mechanism, making it thus impossible to get a complete and accurate image of the biological activities with dual-use potential. A useful step would be to use the already existing registry for HGE preclinical and clinical trials set up by the WHO by broadening its scope to include all genome editing activities and making registration compulsory. A verification mechanism would most likely be more achievable than global regulation which would impose restrictions. As in most other high-stake fields (chemical, nuclear, missiles), international verification of genome editing activities would rely on the “mutual benefit” principle and might be considered a win-win strategy if the stakes are high enough. This would not necessarily start with a legally binding mechanism, but a “soft law” instrument such as a code of conduct (Marchant 2021).

However, we emphasize that a “traditional” verification system for genome editing is unlikely to work, and would more likely take the form of bio-surveillance carried out by the agencies and departments involved in national security, such as gathering, analysing, and interpreting data (on genome editing activities) in order to achieve threat awareness, prevention and early detection (The White House 2007). International cooperation will still be essential, since it will require creating cross-border information-sharing and communication networks (Ostfield 2009; Kosal 2020), and international investigation and detection capabilities.

4. Tracking genome editing technologies

Alternatively, also by taking inspiration from the nuclear field, start to identify supply chain chokepoints to be closely monitored (Kaminska 2021). For example, the nuclear materials, equipment, and technologies supplied on the international market are strictly controlled by the Nuclear Suppliers Group (NSG), thus ensuring that states do not deviate from the peaceful uses of nuclear energy (NSG n.d.). At the moment, the only structure resembling a chain checkpoint for genome editing is the international patent system which manages the use of certain genome editing technologies. Although an arrangement such as the NSG may sound similar to using the international patent system to ensure the peaceful use of genome editing technologies, there is one major difference: in the international patent system the ethical and legal decisions lie in the hands of one or very few stakeholders who own exclusive rights, whereas in NSG or a similar arrangement, the moral and legal decision is through the vote of the majority of all states that use similar technologies. Several solutions to strengthen the patent system have been suggested, such as building on the U.S. 1954 Atomic Energy Act, which deemed non-patentable all technology that would be useful only in the production of fissionable material or, in other words, weaponized (Parthasarathy 2018). The international patent system has indeed great potential when it comes to emerging technological inventions, but it is far from being a one-size-fits-all solution in the control of technology, especially in terms of verification of genome editing activities and control of non-patented genome editing technologies.

## Conclusion

Genome editing has had a major impact on the way biosecurity plays out domestically and internationally from four perspectives: 1) it is making it easier to develop more advanced biological weapons (Esvelt and Millett 2017), 2) it can intentionally or unintentionally be used to damage large ecosystems (e.g.: agro-terrorism or an accidentally escaped virus), 3) it can alter the makeup of armies, and 4) it can (currently) avoid all measures of non-proliferation.

Once new knowledge comes out of the box, it can never be put back, and, as with the discovery of radiation, it is very unlikely that someone out there—a state, a company, or an individual—is not unscrupulously experimenting with genome editing tools already. The fact that we have not yet heard of biosecurity incidents involving genome editing should not appease us, but worry us, and reinforce calls for global governance and oversight. The lack of proper preventive measures through regulation and verification will force us into reacting, rather than acting once we are faced with such an incident. The recent COVID-19 pandemic gave us a bitter taste of the implications of an accidental bio-threat, and if we throw genome editing into the equation, the effects could be catastrophic.

## Data Availability

Not applicable.

## Footnotes

1 For news article on it: https://sciencebusiness.net/news/dual-use/eu-commission-launches-bid-expand-funding-dual-use-research-horizon-europes-successor. For the white paper itself: https://research-and-innovation.ec.europa.eu/system/files/2024-01/ec_rtd_white-paper-dual-use-potential.pdf

2 Perhaps one could suggest that A.I. would be increasingly used in such cases, avoiding all such human problems (and any need for genetic interventions). However, it is not clear that this would be less ethically fraught.
